# Endothelium-Dependent Hyperpolarization (EDH) in Hypertension: The Role of Endothelial Ion Channels

**DOI:** 10.3390/ijms19010315

**Published:** 2018-01-21

**Authors:** Kenichi Goto, Toshio Ohtsubo, Takanari Kitazono

**Affiliations:** Department of Medicine and Clinical Science, Graduate School of Medical Sciences, Kyushu University, Fukuoka 812-8582, Japan; tohtsubo@intmed2.med.kyushu-u.ac.jp (T.O.); kitazono@intmed2.med.kyushu-u.ac.jp (T.K.)

**Keywords:** Ca^2+^-activated K^+^ channel, Ca^2+^-activated Cl^−^ channel, endothelial function, endothelium-dependent hyperpolarization, endothelium-derived hyperpolarizing factor, hypertension, transient receptor potential vanilloid type 4 channel

## Abstract

Upon stimulation with agonists and shear stress, the vascular endothelium of different vessels selectively releases several vasodilator factors such as nitric oxide and prostacyclin. In addition, vascular endothelial cells of many vessels regulate the contractility of the vascular smooth muscle cells through the generation of endothelium-dependent hyperpolarization (EDH). There is a general consensus that the opening of small- and intermediate-conductance Ca^2+^-activated K^+^ channels (SK_Ca_ and IK_Ca_) is the initial mechanistic step for the generation of EDH. In animal models and humans, EDH and EDH-mediated relaxations are impaired during hypertension, and anti-hypertensive treatments restore such impairments. However, the underlying mechanisms of reduced EDH and its improvement by lowering blood pressure are poorly understood. Emerging evidence suggests that alterations of endothelial ion channels such as SK_Ca_ channels, inward rectifier K^+^ channels, Ca^2+^-activated Cl^−^ channels, and transient receptor potential vanilloid type 4 channels contribute to the impaired EDH during hypertension. In this review, we attempt to summarize the accumulating evidence regarding the pathophysiological role of endothelial ion channels, focusing on their relationship with EDH during hypertension.

## 1. Introduction

Endothelial cells play a critical role in the regulation of vascular tone through the release of several vasorelaxing and vasoconstricting factors [1]. In addition to the release of relaxing factors such as nitric oxide (NO) and prostaglandins, endothelial cells relax the vascular smooth muscle cells through the generation of smooth muscle hyperpolarization in an endothelium-dependent manner [2,3,4]. Although the mechanisms by which endothelial cells produce smooth muscle hyperpolarization may vary depending on the vascular beds and species, both diffusible factors and contact-mediated pathways contribute to the endothelium-dependent smooth muscle hyperpolarization [5,6,7].

**Figure 1:**
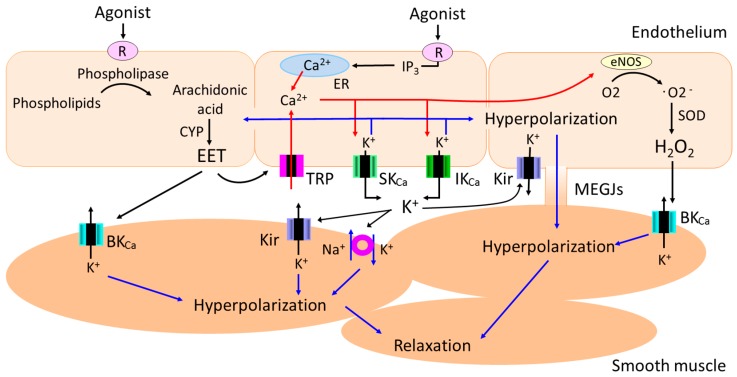
Diffusible and contact-mediated mechanisms of endothelium-dependent smooth muscle hyperpolarization. In certain vascular beds in specific conditions, diffusible factors such as epoxyeicosatrienoic acids (EETs), K^+^ ions, and hydrogen peroxide (H_2_O_2_) hyperpolarize smooth muscle cells through the opening of potassium channels and/or Na^+^/K^+^-ATPase. In addition, endothelium-dependent hyperpolarization initiated in endothelial cells with a rise in intracellular calcium and the subsequent activation of small (SK_Ca_) and intermediate conductance (IK_Ca_) Ca^2+^-activated K^+^ channels spreads to adjacent smooth muscle cells via myoendothelial gap junctions (MEGJs) in a number of vascular beds. In some vascular beds, combination of diffusible and contact-mediated mechanisms underpin smooth muscle hyperpolarization.

In certain vascular beds and specific conditions, diffusible factors such as epoxyeicosatrienoic acids (EETs) [8,9], K^+^ ions [10], C-type natriuretic peptide [11], hydrogen peroxide (H_2_O_2_) [12] and hydrogen sulfide (H_2_S) [13] function as endothelium-derived hyperpolarizing factors (EDHFs). For instance, EETs (which play an important role in the regulation of vascular tone, hemostasis, and inflammation [14,15,16]) released from the endothelial cells transfer to the adjacent smooth muscle cells and produce smooth muscle hyperpolarization through the opening of large conductance Ca^2+^-activated K^+^ (BK_Ca_) channels (Figure 1).

Endothelial cells also produce smooth muscle hyperpolarization in a contact-dependent manner [5,6,7]. Specifically, endothelium-dependent hyperpolarization (EDH) initiated in endothelial cells with a rise in intracellular calcium and the subsequent activation of small (SK_Ca_) and intermediate conductance (IK_Ca_) Ca^2+^-activated K^+^ channels spreads to adjacent smooth muscle cells via myoendothelial gap junctions (MEGJs) in a number of vascular beds [17,18,19,20,21,22,23,24,25]. Although there is a consensus that the intracellular release of Ca^2+^ from the endoplasmic reticulum (ER) and subsequent activation of the SK_Ca_ and IK_Ca_ channels in the endothelium is a prerequisite for the generation of EDH [5,6,7], several studies suggest that Ca^2+^ influx through endothelial non-selective cation channels of the transient receptor potential (TRP) family also plays an important role in EDH via the downstream activation of SK_Ca_ and IK_Ca_ channels in some vascular beds [26,27,28,29,30]. In certain vascular beds in specific conditions, diffusible factors such as EETs and K^+^ ions generate EDH through the activation of endothelial TRP channels [14] and endothelial inward rectifier K^+^ (Kir) channels [31], respectively (Figure 1). As EDH plays a dominant role in endothelium-dependent relaxation in resistance arteries [32,33,34], and pressure in resistance arteries contribute substantially to total peripheral resistance, alterations in the EDH pathway would contribute not only to endothelial function but also to the regulation of arterial blood pressure.

Hypertension is the most important risk factor for cardiovascular disease across the world [35]. Prolonged hypertension causes vascular endothelial dysfunction, which in turn facilitates the progress of atherosclerosis, finally leading to cardiovascular disease [36,37,38]. It is therefore of great importance to uncover the responsible mechanisms and find effective treatments for endothelial dysfunction during hypertension. Although a reduction in NO bioavailability and an enhanced production of endothelium-derived contracting factors contribute to the endothelial dysfunction during hypertension [36,37,39], we have shown that the impaired EDH-mediated hyperpolarization and relaxation contribute to the endothelial dysfunction in mesenteric arteries of spontaneously hypertensive rats (SHR) [40,41]—the most commonly used animal model for human essential hypertension [42]. Reduced EDH during hypertension has also been reported in other models of hypertension [41].

However, the precise mechanisms by which prolonged hypertension impairs EDH-mediated responses are not fully understood. The change in the number and/or function of MEGJs does not seem to be a major contributing factor to impaired EDH-mediated responses during hypertension: first, because previous studies did not observe a positive correlation between EDH-mediated responses and the number of MEGJs in arteries of SHR [43,44]; second, smooth muscle hyperpolarization to 1-EBIO, an endothelial IK_Ca_ channel activator [45], was not altered in mesenteric arteries of SHR, indicating that electrical propagation from endothelial cells to smooth muscle cells via MEGJs is preserved during hypertension [44,46].

Vascular endothelial cells express various types of functional ion channels, including SK_Ca_ channels, IK_Ca_ channels, inward rectifier K^+^ (Kir) channels, ATP-sensitive K^+^ (K_ATP_) channels, voltage-gated K^+^ (Kv) channels, Ca^2+^-activated Cl^−^ channels (CaCCs), and TRP channels [47,48,49]. Alterations of ion channels in vascular endothelial cells during hypertension have been reported [50], and several studies suggest that alterations of the function and/or expression of endothelial ion channels underpin the impaired EDH in animal models of hypertension [46,51,52,53]. In addition, deletion of some of these endothelial ion channels impairs EDH-mediated responses and increases systemic blood pressure in genetically modified mice [54,55].

These findings strongly suggest that alterations of endothelial ion channels greatly contribute to the impaired EDH-mediated responses during hypertension. Such alterations would represent potential therapeutic targets for the prevention of the endothelial dysfunction associated with hypertension. In this review, we discuss the accumulating evidence regarding alterations of endothelial ion channels, focusing on these channels’ relationship with reduced EDH during hypertension.

## 2. Endothelium-Dependent Hyperpolarization (EDH) in Animal Models of Hypertension

In 1992, Van de Voorde et al. revealed that endothelium-dependent hyperpolarization in response to acetylcholine (ACh), which appears to be mediated by the opening of K_Ca_ channels, is impaired in the aorta from two-kidney, one-clip renal hypertensive rats, and they noted that this impairment contributes to the reduced endothelium-dependent relaxation in this model [56]. However, because their study was conducted without inhibiting the synthesis of NO and prostaglandins (both of which produce endothelium-dependent smooth muscle hyperpolarization in certain vascular beds [57,58]), it was difficult to accurately evaluate the relative contribution of EDH to impaired ACh-induced hyperpolarization during hypertension in their rat hypertension model. In a study using the superior mesenteric arteries of SHR, Fujii et al. demonstrated that ACh-induced, endothelium-dependent hyperpolarization and relaxation resistant to inhibitors of NO and prostaglandin synthesis (and thus EDH-mediated responses), were decreased in SHR compared with normotensive Wistar-Kyoto (WKY) rats [40]. Such alterations in EDH during hypertension appear to be secondary to hypertension because EDH-mediated responses are preserved at the pre-hypertensive stage of genetically hypertensive rats [46,59]. Subsequent studies similarly observed impaired EDH-mediated responses in mesenteric [41,46,52,59,60,61,62], renal [63,64,65], coronary [66], femoral [67], and ocular ciliary [68] arteries of genetically hypertensive rats. Reduced EDH-mediated responses have also been reported in arteries from different types of hypertensive rats such as deoxycorticosterone acetate (DOCA)-salt-induced [69,70] and angiotensin II-induced hypertensive rats [71].

In some circumstances, in particular, when NO-mediated vasorelaxation is compromised, EDH may function as a back-up system to maintain overall endothelial function [72]. Indeed, upregulation of EDH-mediated responses in conjunction with reduced NO-mediated responses has been reported in certain vascular beds from animal models of hypertension [73,74,75,76,77]. Further support for this notion is provided by the observation that EDH-mediated responses are upregulated in endothelial nitric oxide synthase (eNOS) knockout mice [78].

The mechanism by which NO inhibits EDH activity remains an open question. However, several possible mechanisms have been suggested including inhibition of cytochrome P-450 enzyme activity by NO [72], reduced permeability of gap junctions by NO [79], and an inhibitory effect of NO on Ca^2+^-permeable nonselective cation channels via a protein kinase G (PKG)-dependent phosphorylation pathway [80]. Nevertheless, it was reported that such an upregulation of EDH during hypertension disappeared when hypertension was sustained over a long period of time [65,81]. Thus, in general, prolonged hypertension produces an impairment of EDH, and this impairment appears to underpin the endothelial dysfunction associated with hypertension. In the following sections, we summarize the ionic mechanisms in vascular endothelial cells contributing to the reduced EDH during hypertension.

## 3. Role of Endothelial Ion Channels in Reduced EDH during Hypertension

### 3.1. Ca^2+^-Activated K^+^ (K_Ca_) Channels

In vascular endothelial cells, both small and intermediate conductance Ca^2+^-activated K^+^ channels (SK_Ca_ and IK_Ca_) are expressed [47,48], and there is a consensus that the activation of the SK_Ca_ and IK_Ca_ channels in the endothelium results in the generation of EDH in a number of vascular beds [5,6,7]. Indeed, the involvement of both SK_Ca_ and IK_Ca_ channels in the generation of EDH has now been supported on the basis of the results from mice deficient in these channels [54,55,82]. In addition, K_Ca_ channel-deficient mice show high blood pressure, suggesting that endothelial K_Ca_ channels play an important role in blood pressure regulation as well [54,55,82]. Although large conductance Ca^2+^-activated K^+^ (BK_Ca_) channels are present in the endothelial cells of some vascular beds [83], there is little evidence showing the involvement of endothelial BK_Ca_ channels in the generation of EDH; or of the presence of endothelial BK_Ca_ channels in intact vessels [5,7].

Changes in the function and/or expression of endothelial SK_Ca_ and IK_Ca_ channels during hypertension, in particular, those of SK_Ca_ channels, have been reported in various types of animal models of hypertension. Thus in mesenteric arteries of SHR and stroke-prone spontaneously hypertensive rats (SHRSP), the function and/or expression of SK_Ca_ channels are reduced and such reduction appears to underpin the impaired EDH-mediated responses in this vascular bed [46,52,84,85]. A contribution of reduced SK_Ca_ channels’ function and/or expression to impaired EDH-mediated responses has also been suggested in mesenteric arteries from angiotensin II-induced hypertensive rats [86], testosterone-induced hypertensive rats [87], and endothelial connexin40 mutant mice that exhibit hypertension [88].

By contrast, preserved [46,86,87,88] or even enhanced [77,85] function and/or expression of IK_Ca_ channels have been reported in hypertensive rats and mice. The reason why SK_Ca_ and IK_Ca_ channels are differentially regulated during hypertension is not clear. The preserved or enhanced function and/or expression of IK_Ca_ channels might be due to the downregulation of the repressor element 1-silencing transcription factor (a negative regulator of IK_Ca_ channels) during hypertension, as has been suggested in mesenteric arteries of SHRSP [85]. In addition, the differential regulation of the two endothelial K_Ca_ channels might be related to the difference in the subcellular localization of the two channels, i.e., SK_Ca_ channels are localized to the endothelial cell membrane (in particular to adjacent endothelial cell borders where connexins, the proteins that compose gap junctions, are also abundantly expressed [89,90,91]), whereas IK_Ca_ channels are, in general, localized to myoendothelial projections which bring endothelial cells into contact with smooth muscle cells through the internal elastic lamina [91,92]. Further studies are required to understand the molecular mechanisms underlying these alterations of endothelial K_Ca_ channels during hypertension.

Although these observations suggest a causative relationship between a reduction in SK_Ca_ channels and the impairment of EDH during hypertension, our recent study indicates that the reduction in SK_Ca_ channels alone is not sufficient to explain the impaired EDH in superior mesenteric arteries of SHRSP [46]. In that study, we observed a significant reduction in SK_Ca_ but not IK_Ca_ channels’ function and expression in superior mesenteric arteries of SHRSP in which the ACh-induced, EDH-mediated responses were substantially impaired compared to those of WKY rats [46]. However, we also found that the EDH-type relaxations induced by simultaneous activators of SK_Ca_ and IK_Ca_ channels (NS309 or SKA-31) did not differ significantly between the two rat strains, suggesting that the preserved function of IK_Ca_ channels together with residual SK_Ca_ channels may help maintain EDH-type relaxation in this vascular bed [46]. Indeed, this prediction is in agreement with the observation that the blockade of SK_Ca_ channels with the selective inhibitor apamin only marginally inhibited ACh-induced, EDH-mediated responses in rat mesenteric arteries [46,93]. These findings indicate that, at least in rat mesenteric arteries, mechanisms other than a reduction in SK_Ca_ channels would also contribute to the impaired EDH during hypertension, as discussed in the following sections.

### 3.2. Transient Receptor Potential (TRP) Channels

Changes in the membrane potential evoked by EDH are composed of two phases: an initial rapid phase followed by a sustained phase [94,95]. The initial rapid phase appears to be mediated by the Ca^2+^ released from intracellular Ca^2+^ stores, and the sustained phase seems to be due to the Ca^2+^ influx through the opening of nonselective cation channels activated by intracellular Ca^2+^ depletion [94,95]. Interestingly, several studies demonstrated that Ca^2+^ influx through endothelial nonselective cation channels of the transient receptor potential (TRP) family plays a critical role in EDH generation in certain vascular beds [26,27,28,29,30].

**Figure 2:**
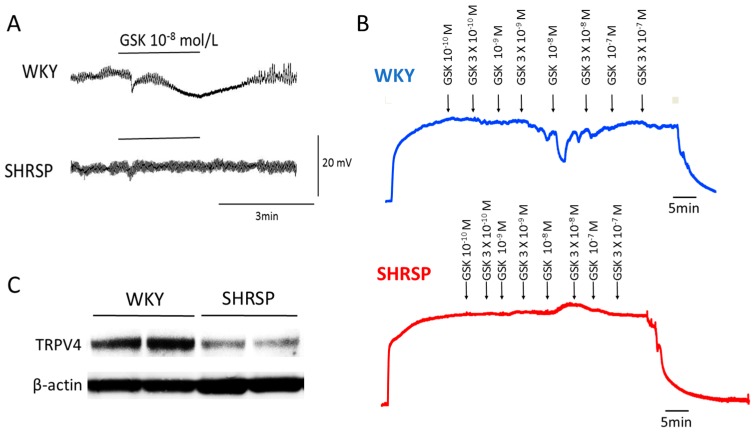
Downregulation of transient receptor potential vanilloid type 4 channel (TRPV4) in hypertension. Representative tracing of GSK1016790A (GSK), a selective TRPV4 activator, induced hyperpolarization (**A**) and relaxation (**B**) in mesenteric arteries of Wistar–Kyoto (WKY) rats and stroke-prone spontaneously hypertensive rats (SHRSP). GSK evoked hyperpolarization and relaxation in WKY but not in SHRSP arteries. Arteries were depolarized (**A**) or pre-contracted (**B**) with phenylephrine (10^−5^ mol/L). Indomethacin (10^−5^ mol/L) and N^ω^-nitro-l-arginine (l-NAME, 10^−4^ mol/L) were present throughout the experiments. (**C**) Representative immunoblots of the expression of TRPV4 in mesenteric arteries from WKY and SHRSP. The expression of the TRPV4 protein was significantly decreased in the SHRSP mesenteric arteries compared with that from WKY. Modified from Seki et al. [46].

Although limited information is currently available about the role of TRP channels in altered EDH-mediated responses during hypertension, several recent studies shed light on the endothelial TRP vanilloid type 4 (TRPV4) channels as a potential target for this alteration. We recently demonstrated that the opening of endothelial TRPV4 channels and the downstream activation of SK_Ca_ and IK_Ca_ are involved in ACh-induced, EDH-mediated responses in superior mesenteric arteries of rats [46]. Our study further demonstrated that endothelial TRPV4 and SK_Ca_ channels are downregulated in SHRSP but not in WKY rats, contributing to the reduced EDH-mediated responses in superior mesenteric arteries of SHRSP [46] (Figure 2). The downregulation of endothelial TRPV4 seems to be secondary to hypertension because the function and expression of TRPV4 were preserved in pre-hypertensive SHRSP [46].

A decrease in the expression of endothelial TRPV4 and a concomitant impairment of endothelium-dependent relaxation has also been reported in mesenteric arteries of SHR [96]. It thus seems likely that a downregulation of endothelial TRPV4 underpins the impaired EDH in genetically hypertensive rats. Indeed, the causative link between the loss of TRPV4 and the impairment of EDH-mediated responses has been observed in mesenteric arteries of TRPV4 knockout mice [97]. Although another member of the TRP channel family, TRP canonical type 3 (TRPC3), has also been shown to be critical for EDH-mediated responses in rat third-order branch of the superior mesenteric arteries [29], we did not detect any alterations of the function or expression of TRPC3 in superior mesenteric arteries of SHRSP [46].

In contrast, in mesenteric resistance arteries of Wistar rats fed a high-salt diet, the expression of TRPV4 was upregulated despite the significant increase in arterial blood pressure [98]. The reason for the discrepancy between the two studies with respect to the expression of TRPV4 during hypertension is not known. One possible explanation is that the upregulation of TRPV4 may be a compensatory mechanism to maintain endothelial function in salt-induced hypertension. This scenario agrees well with the studies demonstrating that a high-salt diet upregulates EDH to compensate for the loss of NO in mesenteric arteries of rats [74,76].

In the myoendothelial projections (MEPs) of mouse mesenteric arteries, the muscarinic receptor activation of TRPV4 is achieved through the following signaling cascade: muscarinic receptors/diacylglycerol/protein kinase C (PKC) and the PKC anchoring protein A-kinase anchoring protein 150 (AKAP150)/TRPV4 channels, where the AKAP150-dependent clustering of TRPV4 channels plays a critical role in the muscarinic receptor activation of TRPV4 [53]. Moreover, in mesenteric arteries of angiotensin II-induced hypertensive mice, this signaling cascade was dysfunctional due to a loss of AKAP150 at MEPs, resulting in reduced EDH-mediated relaxation [53]. The current density of TRPV4 measured by the patch-clamp technique did not differ in the mesenteric endothelial cells of normotensive versus angiotensin II-induced hypertensive mice, indicating that the function and channel numbers of TRPV4 in endothelial cells per se were similar between normotensive and angiotensin II-induced hypertensive mice [53]. Another study also reported that the protein expression of TRPV4 did not differ in mesenteric arteries of normotensive versus angiotensin II-induced hypertensive mice [99]. Taken together, these findings suggest that a reduced expression of AKAP150 at MEPs underpins impaired EDH in mesenteric arteries of angiotensin II-induced hypertensive mice.

More recently, it was reported that an impairment of the physical and functional interaction of TRPV4-SK_Ca_ channels underlies the reduced EDH-mediated responses in small arteries from mice with hypertension induced by a high-salt diet [100]. Of particular interest is that both endothelial SK_Ca_ and TRPV4 channels seem to be compromised during hypertension because these two channels are preferentially localized in caveolae, which are specialized lipid rafts on which a number of transduction proteins are located [101]. The issue of whether or not the loss of endothelial SK_Ca_ and TRPV4 channels during hypertension is associated with any changes in caveolae warrants further investigation.

### 3.3. Inward Rectifier K^+^ (Kir) Channels

A modest increase in the extracellular K^+^ induces vasodilation through the activation of Na^+^/K^+^ ATPase and/or inward rectifier K^+^ (Kir) channels [102]. Although a few studies have shown impaired K^+^-induced vasodilator responses in arteries from animal models of hypertension [103,104] and patients with essential hypertension [105], the underlying mechanisms remain poorly understood. 

In blood vessels, Kir channels are expressed in both smooth muscle cells and endothelial cells [106,107,108]. However, the specific cellular location of Kir channels appears to differ depending on the vascular beds and vessel size involved [31,107]. In mesenteric resistance arteries of rats and mice, several studies have shown that functional Kir channels are localized to the endothelium [90,108,109,110,111,112]. The endothelial Kir channels are activated not only by extracellular K^+^ but also by shear stress [112]. Thus, the absence of endothelial Kir channel activation could increase peripheral resistance and hence contribute to blood pressure elevation. In fact, Kir2.1^+/−^ mice showed increased mean blood pressure probably due to the increased microvascular resistance [112].

We have shown that in mesenteric resistance arteries of WKY rats, after the blockade of NO and prostanoids, the overall hyperpolarization to iontophoresed ACh mediated by EDH was significantly reduced by the inhibition of endothelial Kir channels with barium, whereas barium was without effect in SHR arteries [90]. These findings suggest that ACh-induced EDH is augmented by the activation of endothelial Kir channels in WKY but not in SHR mesenteric resistance arteries. The lack of endothelial Kir channels’ involvement in ACh-induced EDH in the SHR in that study does not appear to be related to the alteration of the function or expression of endothelial Kir channels per se, because barium-sensitive hyperpolarizing responses to exogenously applied K^+^ and the mRNA expression of the Kir2.1 gene in mesenteric resistance arteries did not differ between the WKY rats and SHR [90].

Unaltered K^+^-induced vasodilator responses have also been reported in mesenteric arteries of angiotensin II-induced hypertensive mice [111] and in cortical parenchymal arterioles of SHR [113], where endothelial Kir2.1 channels play a dominant role in K^+^-induced vasodilation [111,114]. In addition, in the smooth muscle cells from mesenteric arteries, the protein expression level of the Kir2.1 did not differ significantly between normotensive blood pressure (BPN) and hypertensive blood pressure (BPH) mice [115]. Together, the above-described findings suggest that the function and expression of endothelial Kir channels per se appear to be preserved during hypertension. Thus, the underlying mechanism of the lack of endothelial Kir channels’ involvement in ACh-induced EDH in mesenteric resistance arteries of the SHR in our study is yet to be determined. Nevertheless, ACh-evoked depolarization mediated by the opening of endothelial Ca^2+^-activated Cl^−^ channels (CaCCs) may result in a failure to activate endothelial Kir channels in this vascular bed [90,116]. This scenario is further supported by the observations that the inhibition of Cl^−^ channels potentiates the EDH-mediated relaxation through the activation of Kir channels in rat mesenteric arteries [108,117].

By contrast, Weston et al. reported that a functional loss of Kir channels in mesenteric resistance arteries of SHR is due to a significant downregulation in the expression of Kir2.1 protein [52]. The discrepancy between the two studies of Kir channel expression cannot be attributable to the difference in the vessel size or the age of the animals used, because the two studies were conducted in mesenteric resistance arteries of rats at about the same age (12 weeks in the Goto et al. study vs. 12–16 weeks in the Weston et al. study) [52,90]. The difference in the expression of Kir channels might be due to the difference in the systolic blood pressure levels of the SHR used (199 ± 5 mmHg in the Goto et al. study vs. 235 ± 5 mmHg in the Weston et al. study) [52,90]. Further studies are required to elucidate the mechanisms underlying the lack of endothelial Kir channels involvement in EDH in the mesenteric resistance arteries of SHR.

### 3.4. Voltage-Gated K^+^ (Kv) Channels and ATP-Sensitive K^+^ (K_ATP_) Channels

Voltage-gated K^+^ (Kv) channels are activated by membrane depolarization so that the resting membrane potential becomes more negative, and thus these channels contribute to the regulation of vascular smooth muscle tone [118]. Kv channels are highly expressed in vascular smooth muscle cells, and a number of studies have reported the reduced function and expression of smooth muscle Kv channels during hypertension [119]. Several studies have suggested the expression of Kv channels in vascular endothelial cells; however, the physiological role of endothelial Kv channels is poorly understood [48]. The involvement of endothelial Kv channels in EDH generation in guinea-pig carotid artery endothelial cells has been suggested [120]. Endothelial cells of the rat aorta from SHRSP have been reported to show lower levels of Kv1.5 function and expression compared to those from WKY rats [121]. However, it is not yet known whether a decreased expression of endothelial Kv channels contributes to the impaired EDH during hypertension.

ATP-sensitive K^+^ (K_ATP_) channels are abundantly expressed in vascular smooth muscle cells [106], and several studies have reported the reduced function of K_ATP_ channels during hypertension [122,123]. The expression of endothelial K_ATP_ channels was also reported in some vascular beds [48]. Although endothelial K_ATP_ channels appear to contribute to vasodilation induced by hyperosmolality [124] or hypoxia [125], it is not known whether endothelial K_ATP_ channels contribute to EDH and whether function and expression of endothelial K_ATP_ channels are altered during hypertension.

As stated above, little is currently known about the role of endothelial Kv and K_ATP_ channels in the regulation of EDH during hypertension. Nevertheless, it has been shown that both H_2_O_2_ [126,127] and H_2_S [13,128] produce hyperpolarization of the vascular smooth muscle cells through the activation of Kv and/or K_ATP_ channels in certain vascular beds. Interestingly, in the mesenteric arteries of SHR, relaxation response to exogenously applied H_2_O_2_ is reduced due to an impaired Kv channels activity [129]. In addition, reduction in endogenous H_2_S levels and H_2_S-induced vasorelaxation have been shown in animal models of hypertension as well as hypertensive patients [130,131]. Whether or not endothelial Kv and/or K_ATP_ channels contribute to the impaired EDH during hypertension in arteries where H_2_O_2_ and/or H_2_S act as EDHF requires further investigation.

### 3.5. Ca^2+^-Activated Chloride Channels (CaCCs)

In addition to their expression in vascular smooth muscle cells, Ca^2+^-activated Cl^−^ channels (CaCCs) have been shown to be present in vascular endothelial cells [47]. Because the resting membrane potential of vascular smooth muscle cells is more negative than the reversal potential for chloride ion (Cl^−^), the opening of Cl^−^ channels leads to an efflux of Cl^−^ ions and depolarizes the membrane potential [132]. The activation of endothelial CaCCs would also evoke membrane depolarization, and the depolarization could counteract EDH induced by the activation of endothelial SK_Ca_ and IK_Ca_ channels because the vascular smooth muscle and endothelial cells behave as an electrical syncytium due to the presence of gap junctions [133]. Indeed, the effects of CaCCs have been suggested to counteract those of K_Ca_ channels in vascular endothelial cells [134,135,136]. 

Several studies identified that the transmembrane member 16A (TMEM16A; also known as Ano1) protein is a molecular counterpart for CaCCs [137,138,139]. In arteries of SHR, the TMEM16A expression level is increased and the systemic blockade of TMEA16A by peripheral administration of a TMEA16A inhibitor, T16Ainh-A01, reduced blood pressure in this model [140]. In angiotensin II-induced hypertensive mice, the knockout of endothelial-specific TMEM 16A lowered the systolic blood pressure and ameliorated endothelial function, whereas the overexpression of endothelial-specific TMEM 16A elevated the systolic blood pressure and deteriorated endothelial function [141]. These findings suggest that CaCC TMEM 16A located on the endothelium contributes to the impairment of endothelial function and thus blood pressure elevation in hypertension. 

**Figure 3:**
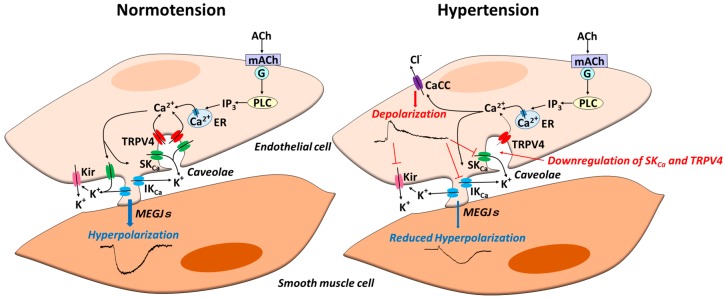
Endothelial ion channels in normotension and hypertension. In normotension, in response to agonist stimulation of endothelial cells, a rise in intracellular Ca^2+^ occurs due to the release from intracellular Ca^2+^ stores and Ca^2+^ entry via transient potential vanilloid type 4 channel (TRPV4). A rise in intracellular Ca^2+^ subsequently generates endothelium-dependent hyperpolarization (EDH) through the activation of both small (SK_Ca_) and intermediate conductance (IK_Ca_) Ca^2+^-activated K^+^ channels. In some arteries, K^+^ released from endothelial K_Ca_ channels activates endothelial Kir channels, which in turn amplifies EDH. EDH spreads to adjacent smooth muscle cells via myoendothelial gap junctions (MEGJs), resulting in vascular relaxation. In hypertension, alterations of endothelial ion channels additively reduce EDH; these alterations include downregulation of endothelial SK_Ca_ and TRPV4 channels, upregulation of endothelial Ca^2+^-activated chloride channels (CaCCs), and functional loss of endothelial Kir channels.

In mesenteric resistance arteries of SHR, after the blockade of EDH with K_Ca_ channel inhibitors, iontophoresed ACh evoked an endothelium-dependent depolarization through the opening of CaCCs that was effectively absent in WKY arteries [116]. In that study, the inhibition of the ACh-evoked depolarization by CaCC inhibitors improved the ACh-induced EDH, suggesting that the upregulation of endothelial CaCCs at least partly underpins the impaired EDH in the mesenteric arteries of SHR [116] (Figure 3). Although the molecular identity for the CaCC observed in that study was unknown at the time [116], the subsequent findings described above strongly suggest that TMEM 16A is the molecular counterpart for CaCCs in mesenteric arteries of SHR. Thus, the impact of endothelial CaCC TMEM 16A on altered EDH during hypertension and therefore on blood pressure regulation warrants further investigation.

## 4. Therapeutic Implications

A number of studies (including our group’s) have demonstrated that antihypertensive treatment improves the impairment of EDH-mediated responses associated with hypertension [41,61,142,143,144,145,146,147]. As several classes of antihypertensive drugs such as angiotensin-converting enzyme (ACE) inhibitors [61,142,143,144,145,146], angiotensin II type 1 receptor blockers [144,145,147], angiotensin II receptor–neprilysin inhibitor [147], and diuretics [143] were effective in improving reduced EDH-mediated responses associated with hypertension, blood pressure lowering per se appears to play a crucial role in these improvements. However, the inhibition of renin–angiotensin system (RAS) by a RAS inhibitor may have an additional benefit against EDH beyond, or in combination with, blood pressure lowering. Indeed, in the mesenteric arteries of WKY rats, treatment with RAS inhibitors but not diuretics prevented the age-related decline in EDH-mediated responses despite similar effects on blood pressure [148,149].

The underlying mechanisms by which antihypertensive treatments improve the reduced EDH-mediated responses associated with hypertension are currently far from clear. In mesenteric arteries of SHR, a reduction of blood pressure with enalapril (an ACE inhibitor) augmented EDH with no significant changes in the endothelial connexin expression or the number of MEGJs, suggesting that an upregulation of gap junctions was not involved in the restoration of EDH in that study [44]. Several studies have demonstrated that ACE inhibitors restore the impairment of EDH-mediated responses through the upregulation of SK_Ca_ and/or IK_Ca_ channels in hypertensive rats [150,151]. In line with these observations, it has been reported that angiotensin II inhibits K_Ca_ channels in isolated vascular smooth muscle cells [152,153].

Another possible explanation is that the improvement of EDH-mediated responses results from the reduced oxidative stress during antihypertensive treatments. This possibility is based on the report that oxidative stress impairs both SK_Ca_ and IK_Ca_ channel functions via a downregulation of these two endothelial K_Ca_ channels in rat mesenteric arteries [154]. However, caution should be exercised in generalizing the results of that report [154], because other studies have demonstrated that EDH-mediated responses are insensitive to oxidative stress in other vascular beds [155,156]. Further studies are needed to determine whether an upregulation of endothelial K_Ca_ channels contributes to the improvement of EDH-mediated responses during antihypertensive treatments, and if so, to elucidate the mechanisms underlying the regulation of endothelial K_Ca_ channels in hypertension.

## 5. Endothelium-Dependent Hyperpolarization (EDH) in Human Hypertension

As discussed in the preceding paragraphs, EDH-mediated responses are reduced in animal models of hypertension. However, caution should be exercised in extrapolating data from animals to humans. Although many studies mentioned above were conducted using SHR strain, it has been suggested that this strain has significant genetic diversity between laboratories [157]. Such genetic diversity might cause difference in EDH-mediated responses independently of blood pressure. Caution should also be taken when interpreting the role of ion channels for EDH in genetically manipulated mice because the difference in genetic background and genetic compensation could influence the phenotype [158]. Thus, it is of particular importance to gather human data.

Although the contribution of EDH to the regulation of vasomotor tone has been demonstrated in a number of human arteries [159], only a few studies have investigated the role of EDH in human hypertension. In patients with essential hypertension, the upregulation of EDH-mediated responses compensates for the loss of NO to maintain endothelium-dependent vasodilation in the forearm microcirculation [160,161] and in the small arteries dissected from gluteal biopsies [162].

By contrast, both decreased NO-mediated and decreased EDH-mediated responses have been reported in the great omental arteries of essential hypertensive patients [163]. Decreased EDH-mediated responses have also been shown in small myometrial arteries from patients with preeclampsia, a condition in pregnancy characterized by hypertension and proteinuria [164,165,166]. The compromised myoendothelial communication [165] and/or downregulation of endothelial IK_Ca_ channels due to the nicotinamide adenine dinucleotide phosphate (NADPH) oxidase 2-derived superoxide [167] may be, at least in part, associated with impaired EDH-mediated responses in patients with preeclampsia. Although treatment with ACE inhibitors improved endothelial function through the upregulation of EDH-mediated responses in the internal thoracic artery of hypertensive patients [168], the underlying mechanisms of this improvement are not known.

## 6. Conclusions

Endothelium-dependent hyperpolarization (EDH) and EDH-mediated relaxation are impaired during prolonged hypertension. Accumulating evidence from animal models of hypertension suggests that alterations of endothelial ion channels contribute to the impaired EDH-mediated responses during hypertension. A number of studies have demonstrated that the reduced function and/or expression of endothelial SK_Ca_ channels play a causative role in this impairment. In addition, emerging evidence reveals that the decrease in EDH-mediated responses in hypertension is accompanied by a loss of function and/or expression of endothelial TRPV4 channels. The membrane depolarization evoked by the activation of endothelial CaCCs appears to counteract the hyperpolarizing influence of endothelial K_Ca_ and Kir channels, thereby leading to a reduction in EDH-mediated responses during hypertension. Thus, it is reasonable to suggest that alterations of these endothelial ion channels could have a substantial impact on EDH during hypertension.

Although some studies have suggested a causative relationship between oxidative stress and the loss of endothelial K_Ca_ channels, the mechanisms underlying these alterations of endothelial ion channels during hypertension remain largely unknown. The existence of EDH has been demonstrated in a number of human arteries. Moreover, EDH-mediated responses are decreased in some but not all arteries from patients with hypertension. As EDH is a dominant vasodilator in resistance arteries (that play an important role in blood pressure control), an in-depth understanding of the mechanisms that regulate the alteration of endothelial ion channels during hypertension could pave the way for new treatments for hypertension and related cardiovascular diseases.

## Abbreviations

ACE	Angiotensin converting enzyme
ACh	Acetylcholine
AKAP150	A-kinase anchoring protein 150
BK_Ca_	Large conductance Ca^2+^-activated K^+^
CaCC	Ca^2+^-activated Cl^−^ channel
CYP	Cytochrome P450
DOCA	Deoxycorticosterone acetate
EDH	Endothelium-dependent hyperpolarization
EDHF	Endothelium-derived hyperpolarizing factor
EETs	Epoxyeicosatrienoic acids
eNOS	endothelial nitric oxide synthase
GSK	GSK1016790A
H_2_O_2_	Hydrogen peroxide
H_2_S	Hydrogen sulfide
IK_Ca_	Intermediate–conductance Ca^2+^-activated K^+^
K_ATP_	ATP-sensitive K^+^
Kir	Inward rectifier K^+^
Kv	Voltage-gated K^+^
l-NAME	N^ω^-nitro-l-arginine
MEGJs	Myoendothelial gap junctions
MEPs	Myoendothelial projections
NADPH	Nicotinamide adenine dinucleotide phosphate
NO	Nitric oxide
PKC	Protein kinase C
RAS	Renin–angiotensin system
SHR	Spontaneously hypertensive rats
SHRSP	Stroke-prone spontaneously hypertensive rats
SK_Ca_	Small-conductance Ca^2+^-activated K^+^
TMEM16A	Transmembrane member 16A
TRP	Transient receptor potential
TRPC3	TRP canonical type 3
TRPV4	TRP vanilloid type 4
WKY	Wistar-Kyoto

## References

[1] Hill C.E., Phillips J.K., Sandow S.L. (2001). Heterogeneous control of blood flow amongst different vascular beds. Med. Res. Rev..

[2] Feletou M., Vanhoutte P.M. (1988). Endothelium-dependent hyperpolarization of canine coronary smooth muscle. Br. J. Pharmacol..

[3] Chen G., Suzuki H., Weston H.A. (1988). Acetylcholine releases endothelium-derived hyperpolarizing factors and EDRF from rat blood vessels. Br. J. Pharmacol..

[4] Garland C.J., McPherson G.A. (1992). Evidence that nitric oxide does not mediate the hyperpolarization and relaxation to acetylcholine in the rat small mesenteric artery. Br. J. Pharmacol..

[5] Busse R., Edwards G., Feletou M., Fleming I., Vanhoutte P.M., Weston A.H. (2002). EDHF: Bringing the concepts together. Trends Pharmacol. Sci..

[6] Sandow S.L. (2004). Factors, fiction and endothelium-derived hyperpolarizing factor. Clin. Exp. Pharmacol. Physiol..

[7] Garland C.J., Dora K.A. (2017). EDH: Endothelium-dependent hyperpolarization and microvascular signalling. Acta Physiol..

[8] Campbell W.B., Gebremedhin D., Pratt P.F., Harder D.R. (1996). Identification of epoxyeicosatrienoic acids as endothelium-derived hyperpolarizing factors. Circ. Res..

[9] Fisslthaler B., Popp R., Kiss L., Potente M., Harder D.R., Fleming I., Busse R. (1999). Cytochrome P450 2C is an EDHF synthase in coronary arteries. Nature.

[10] Edwards G., Dora K.A., Gardener M.J., Garland C.J., Weston A.H. (1998). K^+^ is an endothelium-derived hyperpolarizing factor in rat arteries. Nature.

[11] Chauhan S.D., Nilsson H., Ahluwalia A., Hobbs A.J. (2003). Release of C-type natriuretic peptide accounts for the biological activity of endothelium-derived hyperpolarizing factor. Proc. Natl. Acad. Sci. USA.

[12] Matoba T., Shimokawa H., Nakashima M., Hirakawa Y., Mukai Y., Hirano K., Kanaide H., Takeshita A. (2000). Hydrogen peroxide is an endothelium-derived hyperpolarizing factor in mice. J. Clin. Investig..

[13] Mustafa A.K., Sikka G., Gazi S.K., Steppan J., Jung S.M., Bhunia A.K., Barodka V.M., Gazi F.K., Barrow R.K., Wang R. (2011). Hydrogen sulfide as endothelium-derived hyperpolarizing factor sulfhydrates potassium channels. Circ. Res..

[14] Campbell W.B., Fleming I. (2010). Epoxyeicosatrienoic acids and endothelium-dependent responses. Pflugers. Arch..

[15] Larsen B.T., Campbell W.B., Gutterman D.D. (2007). Beyond vasodilatation: Non-vasomotor roles of epoxyeicosatrienoic acids in the cardiovascular system. Trends Pharmacol. Sci..

[16] Node K., Huo Y., Ruan X., Yang B., Spiecker M., Ley K., Zeldin D.C., Liao J.K. (1999). Anti-inflammatory properties of cytochrome P450 epoxygenase-derived eicosanoids. Science.

[17] Hashitani H., Suzuki H. (1997). K^+^ channels which contribute to the acetylcholine-induced hyperpolarization in smooth muscle of the guinea-pig submucosal arteriole. J. Physiol..

[18] Crane G.J., Gallagher N., Dora K.A., Garland C.J. (2003). Small- and intermediate-conductance calcium-activated K^+^ channels provide different facets of endothelium-dependent hyperpolarization in rat mesenteric artery. J. Physiol..

[19] Chaytor A.T., Evans W.H., Griffith T.M. (1998). Central role of heterocellular gap junctional communication in endothelium-dependent relaxations of rabbit arteries. J. Physiol..

[20] Yamamoto Y., Imaeda K., Suzuki H. (1999). Endothelium-dependent hyperpolarization and intracellular electrical coupling in guinea-pig mesenteric arterioles. J. Physiol..

[21] Sandow S.L., Hill C.E. (2000). Incidence of myoendothelial gap junctions in the proximal and distal mesenteric arteries of the rat is suggestive of a role in endothelium-derived hyperpolarizing factor-mediated responses. Circ. Res..

[22] Coleman H.A., Tare M., Parkington H.C. (2001). K^+^ currents underlying the action of endothelium-derived hyperpolarizing factor in guinea-pig, rat and human blood vessels. J. Physiol..

[23] Sandow S.L., Tare M., Coleman H.A., Hill C.E., Parkington H.C. (2002). Involvement of myoendothelial gap junctions in the actions of endothelium-derived hyperpolarizing factor. Circ. Res..

[24] Goto K., Fujii K., Kansui Y., Abe I., Iida M. (2002). Critical role of gap junctions in endothelium-dependent hyperpolarization in rat mesenteric arteries. Clin. Exp. Pharmacol. Physiol..

[25] Dora K.A., Sandow S.L., Gallagher N.T., Takano H., Rummery N.M., Hill C.E., Garland C.J. (2003). Myoendothelial gap junctions may provide the pathway for EDHF in mouse mesenteric artery. J. Vasc. Res..

[26] Earley S., Gonzales A.L., Crnich R. (2009). Endothelium-dependent cerebral artery dilation mediated by TRPA1 and Ca^2+^-Activated K^+^ channels. Circ. Res..

[27] Earley S., Gonzales A.L., Garcia Z.I. (2010). A dietary agonist of transient receptor potential cation channel V3 elicits endothelium-dependent vasodilation. Mol. Pharmacol..

[28] Sonkusare S.K., Bonev A.D., Ledoux J., Liedtke W., Kotlikoff M.I., Heppner T.J., Hill-Eubanks D.C., Nelson M.T. (2012). Elementary Ca^2+^ signals through endothelial TRPV4 channels regulate vascular function. Science.

[29] Senadheera S., Kim Y., Grayson T.H., Toemoe S., Kochukov M.Y., Abramowitz J., Housley G.D., Bertrand R.L., Chadha P.S., Bertrand P.P. (2012). Transient receptor potential canonical type 3 channels facilitate endothelium-derived hyperpolarization-mediated resistance artery vasodilator activity. Cardiovasc. Res..

[30] Kochukov M.Y., Balasubramanian A., Abramowitz J., Birnbaumer L., Marrelli S.P. (2014). Activation of endothelial transient receptor potential C3 channel is required for small conductance calcium-activated potassium channel activation and sustained endothelial hyperpolarization and vasodilation of cerebral artery. J. Am. Heart Assoc..

[31] Jackson W.F. (2017). Boosting the signal: Endothelial inward rectifier K^+^ channels. Microcirculation.

[32] Nagao T., Illiano S., Vanhoutte P.M. (1992). Heterogeneous distribution of endothelium-dependent relaxations resistant to NG-nitro-l-arginine in rats. Am. J. Physiol..

[33] Hwa J.J., Ghibaudi L., Williams P., Chatterjee M. (1994). Comparison of acetylcholine-dependent relaxation in large and small arteries of rat mesenteric vascular bed. Am. J. Physiol..

[34] Shimokawa H., Yasutake H., Fujii K., Owada M.K., Nakaike R., Fukumoto Y., Takayanagi T., Nagao T., Egashira K., Fujishima M. (1996). The importance of the hyperpolarizing mechanism increases as the vessel size decreases in endothelium-dependent relaxations in rat mesenteric circulation. J. Cardiovasc. Pharmacol..

[35] Lawers C.M., Hoorn S.V., Rodgers A. (2008). Global burden of blood-pressure-related disease. Lancet.

[36] Vanhoutte P.M. (1997). Endothelial dysfunction and atherosclerosis. Eur. Heart J..

[37] Schiffrin E.L. (2002). Beyond blood pressure: The endothelium and atherosclerosis progression. Am. J. Hypertens..

[38] Halcox J.P., Schenke W.H., Zalos G., Mincemoyer R., Prasad A., Waclawiw M.A., Nour K.R., Quyyumi A.A. (2002). Prognostic value of coronary vascular endothelial dysfunction. Circulation.

[39] Vanhoutte P.M., Feletou M., Taddei S. (2005). Endothelium-dependent contractions in hypertension. Br. J. Pharmacol..

[40] Fujii K., Tominaga M., Ohmori S., Kobayashi K., Koga T., Takata Y., Fujishima M. (1992). Decreased endothelium-dependent hyperpolarization to acetylcholine in smooth muscle of the mesenteric artery of spontaneously hypertensive rats. Circ. Res..

[41] Goto K., Fujii K., Kansui Y., Iida M. (2004). Changes in endothelium-derived hyperpolarizing factor in hypertension and ageing: Response to chronic treatment with renin-angiotensin system inhibitors. Clin. Exp. Pharmacol. Physiol..

[42] Nabika T., Nara Y., Ikeda K., Endo J., Yamori Y. (1991). Genetic heterogeneity of the spontaneously hypertensive rat. Hypertension.

[43] Sandow S.L., Bramich N.J., Bandi H.P., Rummery N.M., Hill C.E. (2003). Structure, function, and endothelium-derived hyperpolarizing factor in the caudal artery of the SHR and WKY rat. Arterioscler. Thromb. Vasc. Biol..

[44] Ellis A., Goto K., Chaston D.J., Brackenbury T.D., Meaney K.R., Falck J.R., Wojcikiewicz R.J., Hill C.E. (2009). Enalapril treatment alters the contribution of epoxyeicosatrienoic acids but not gap junctions to endothelium-derived hyperpolarizing factor activity in mesenteric arteries of spontaneously hypertensive rats. J. Pharmacol. Exp. Ther..

[45] Walker S.D., Dora K.A., Ings N.T., Crane G.J., Garland C.J. (2001). Activation of endothelial cell IK(Ca) with 1-ethyl-2-benzimidazolinone evokes smooth muscle hyperpolarization in rat isolated mesenteric artery. Br. J. Pharmacol..

[46] Seki T., Goto K., Kiyohara K., Kansui Y., Murakami N., Haga Y., Ohtsubo T., Matsumura K., Kitazono T. (2017). Downregulation of endothelial transient receptor potential vanilloid type 4 channel and small-conductance of Ca^2+^-activated K^+^ channels underpins impaired endothelium-dependent hyperpolarization in hypertension. Hypertension.

[47] Nilius B., Droogmans G. (2001). Ion channels and their functional role in vascular endothelium. Physiol. Rev..

[48] Jackson W.F. (2005). Potassium channels in the peripheral microcirculation. Microcirculation.

[49] Earley S., Brayden J.E. (2015). Transient receptor potential channels in the vasculature. Physiol. Rev..

[50] Ohya Y., Fujishima M., Hagen T. (2002). Alterations of ion channels in vascular muscle cells and endothelial cells during hypertension and aging. Advances in Cell. Aging and Gerontology.

[51] Grgic I., Kaistha B.P., Hoyer J., Köhler R. (2009). Endothelial Ca^+^-activated K^+^ channels in normal and impaired EDHF-dilator responses-relevance to cardiovascular pathologies and drug discovery. Br. J. Pharmacol..

[52] Weston A.H., Porter E.L., Harno E., Edwards G. (2010). Important of endothelial SK_Ca_ channels and of downstream hyperpolarizing pathways in mesenteric arteries from spontaneously hypertensive rats. Br. J. Pharmacol..

[53] Sonkusare S.K., Dalsgaard T., Bonev A.D., Hill-Eubanks D.C., Kotlikoff M.I., Scott J.D., Santana L.F., Nelson M.T. (2014). AKAP150-dependent cooperative TRPV4 channel gating is central to endothelium-dependent vasodilation and is disrupted in hypertension. Sci. Signal..

[54] Taylor M.S., Bonev A.D., Gross T.P., Eckman D.M., Brayden J.E., Bond C.T., Adelman J.P., Nelson M.T. (2003). Altered expression of small-conductance Ca^2+^-activated K^+^ (SK3) channels modulates arterial tone and blood pressure. Circ. Res..

[55] Brähler S., Kaistha A., Schmidt V.J., Wölfle S.E., Busch C., Kaistha B.P., Kacik M., Hasenau A.L., Grgic I., Si H. (2009). Genetic deficit of SK3 and IK1 channels disrupts the endothelium-derived hyperpolarizing factor vasodilator pathway and causes hypertension. Circulation.

[56] Van de Voorde J., Vanheel B., Leusen I. (1992). Endothelium-dependent relaxation and hyperpolarization in aorta from control and renal hypertensive rats. Circ. Res..

[57] Tare M., Parkington H.C., Coleman H.A., Neild T.O., Dusting G.J. (1990). Hyperpolarization and relaxation of arterial smooth muscle caused by nitric oxide derived from the endothelium. Nature.

[58] Parkington H.C., Tare M., Tonta M.A., Coleman H.A. (1993). Stretch revealed three components in the hyperpolarization of guinea-pig coronary artery in response to acetylcholine. J. Physiol..

[59] Fujii K., Ohmori S., Tominaga M., Abe I., Takata Y., Ohya Y., Kobayashi K., Fujishima M. (1993). Age-related changes in endothelium-dependent hyperpolarization in the rat mesenteric artery. Am. J. Physiol..

[60] Mantelli L., Amerini S., Ledda F. (1995). Roles of nitric oxide and endothelium-derived hyperpolarizing factor in vasorelaxant effect of acetylcholine as influenced by aging and hypertension. J. Cardiovasc. Pharmacol..

[61] Kähönen M., Mäkynen H., Wu X., Arvola P., Pörsti I. (1995). Endothelial function in spontaneously hypertensive rats: Influence of quinapril treatment. Br. J. Pharmacol..

[62] Sunano S., Watanabe H., Tanaka S., Sekiguchi F., Shimamura K. (1999). Endothelium-derived relaxing, contracting and hyperpolarizing factors of mesenteric arteries of hypertensive and normotensive rats. Br. J. Pharmacol..

[63] Hayakawa H., Hirata Y., Suzuki E., Sugimoto T., Matsuoka H., Kikuchi K., Nagano T., Hirobe M., Sugimoto T. (1993). Mechanisms for altered endothelium-dependent vasorelaxation in isolated kidneys from experimental hypertensive rats. Am. J. Physiol..

[64] Dohi Y., Kojima M., Sato K. (1996). Benidipine improves endothelial function in renal resistance arteries of hypertensive rats. Hypertension.

[65] Büssemaker E., Popp R., Fisslthaler B., Larson C.M., Fleming I., Busse R., Brandes R.P. (2003). Aged spontaneously hypertensive rats exhibit a selective loss of EDHF-mediated relaxation in the renal artery. Hypertension.

[66] Vázquez-Pérez S., Navarro-Cid J., de las Heras N., Cediel E., Sanz-Rosa D., Ruilope L.M., Cachofeiro V., Lahera V. (2001). Relevance of endothelium-derived hyperpolarizing factor in the effects of hypertension on rat coronary relaxations. J. Hypertens..

[67] Mori Y., Ohyanagi M., Koida S., Ueda A., Ishiko K., Iwasaki T. (2006). Effects of endothelium-derived hyperpolarizing factor and nitric oxide on endothelial function in femoral resistance arteries of spontaneously hypertensive rats. Hypertens. Res..

[68] Dong Y., Watabe H., Cui J., Abe S., Sato N., Ishikawa H., Yoshitomi T. (2010). Reduced effects of endothelium-derived hyperpolarizing factor in ocular ciliary arteries from spontaneous hypertensive rats. Exp. Eye Res..

[69] Mäkynen H., Kähönen M., Wu X., Arvola P., Pörsti I. (1996). Endothelial function in deoxycorticosterone-NaCl hypertension: Effect of calcium supplementation. Circulation.

[70] Adeagbo A.S., Joshua I.G., Falkner C., Matheson P.J. (2003). Tempol, an antioxidant, restores endothelium-derived hyperpolarizing factor-mediated vasodilation during hypertension. Eur. J. Pharmacol..

[71] Dal-Ros S., Bronner C., Schott C., Kane M.O., Chataigneau M., Schini-Kerth V.B., Chataigneau T. (2009). Angiotensin II-induced hypertension is associated with a selective inhibition of endothelium-derived hyperpolarizing factor-mediated responses in the rat mesenteric artery. J. Pharmacol. Exp. Ther..

[72] Nishikawa Y., Stepp D.W., Chilian W.M. (2000). Nitric oxide exerts feedback inhibition on EDHF-induced coronary arteriolar dilation in vivo. Am. J. Physiol. Heart Circ. Physiol..

[73] Bund S.J. (1998). Influence of mode of contraction on the mechanism of acetylcholine-mediated relaxation of coronary arteries from normotensive and spontaneously hypertensive rats. Clin. Sci..

[74] Sofola O.A., Knill A., Hainsworth R., Drinkhill M. (2002). Change in endothelial function in mesenteric arteries of Sprague-Dawley rats fed a high salt diet. J. Physiol..

[75] Sendao O., Liveira A.P., Bendhack L.M. (2004). Relaxation induced by acetylcholine involves endothelium-derived hyperpolarizing factor in 2-kidney 1-clip hypertensive rat carotid arteries. Pharmacology.

[76] Goto K., Kansui Y., Oniki H., Ohtsubo T., Matsumura K., Kitazono T. (2012). Upregulation of endothelium-derived hyperpolarizing factor compensates for the loss of nitric oxide in mesenteric arteries of dahl salt-sensitive hypertensive rats. Hypertens. Res..

[77] Simonet S., Isabelle M., Bousquenaud M., Clavreul N., Félétou M., Vayssettes-Courchay C., Verbeuren T.J. (2012). KCa 3.1 channels maintain endothelium-dependent vasodilatation in isolated perfused kidneys of spontaneously hypertensive rats after chronic inhibition of NOS. Br. J. Pharmacol..

[78] Waldron G.J., Ding H., Lovren F., Kubes P., Triggle C.R. (1999). Acetylcholine-induced relaxation of peripheral arteries isolated from mice lacking endothelial nitric oxide synthase. Br. J. Pharmacol..

[79] Lu C., McMahon D.G. (1997). Modulation of hybrid bass retinal gap junctional channel gating by nitric oxide. J. Physiol..

[80] Dora K.A., Garland C.J., Kwan H.Y., Yao X. (2001). Endothelial cell protein kinase G inhibits release of EDHF through a PKG-sensitive cation channel. Am. J. Physiol. Heart Circ. Physiol..

[81] Onaka U., Fujii K., Abe I., Fujishima M., Vanhoutt P.M. (1999). Antihypertensive therapy improves endothelium dependent hyperpolarization. Endothelium-Dependent Hyperpolarization.

[82] Köhler R., Ruth P. (2010). Endothelial dysfunction and blood pressure alterations in K^+^-channel transgenic mice. Pflugers. Arch..

[83] Sandow S.L., Grayson T.H. (2009). Limits of isolation and culture: Intact vascular endothelium and BKCa. Am. J. Physiol. Heart Circ. Physiol..

[84] Kong B.W., Man R.Y., Gao Y., Vanhoutte P.M., Leung S.W. (2015). Reduced activity of SKC a and Na-K ATPase underlies the accelerated impairment of EDH-type relaxations in mesenteric arteries of aging spontaneously hypertensive rats. Pharmacol. Res. Perspect..

[85] Giachini F.R., Carneiro F.S., Lima V.V., Carneiro Z.N., Dorrance A., Webb R.C., Tostes R.C. (2009). Upregulation of intermediate calcium-activated potassium channels counterbalance the impaired endothelium-dependent vasodilation in stroke-prone spontaneously hypertensive rats. Transl. Res..

[86] Hilgers R.H., Webb R.C. (2007). Reduced expression of SKCa and IKCa channel proteins in rat small mesenteric arteries during angiotensin II-induced hypertension. Am. J. Physiol. Heart Circ. Physiol..

[87] Chinnathambi V., Yallampalli C., Sathishkumar K. (2013). Prenatal testosterone induces sex-specific dysfunction in endothelium-dependent relaxation pathways in adult male and female rats. Biol. Reprod..

[88] Chaston D.J., Haddock R.E., Howitt L., Morton S.K., Brown R.D., Matthaei K.I., Hill C.E. (2015). Perturbation of chemical coupling by an endothelial Cx40 mutant attenuates endothelium-dependent vasodilation by KCa channels and elevates blood pressure in mice. Pflugers. Arch..

[89] Kansui Y., Fujii K., Nakamura K., Goto K., Oniki H., Abe I., Shibata Y., Iida M. (2004). Angiotensin II receptor blockade corrects altered expression of gap junctions in vascular endothelial cells from hypertensive rats. Am. J. Physiol. Heart Circ. Physiol..

[90] Goto K., Rummery N.M., Grayson T.H., Hill C.E. (2004). Attenuation of conducted vasodilatation in rat mesenteric arteries during hypertension: Role of inwardly rectifying potassium channels. J. Physiol..

[91] Sandow S.L., Neylon C.B., Chen M.X., Garland C.J. (2006). Spatial separation of endothelial small- and intermediate-conductance calcium-activated potassium channels (K(Ca)) and connexins: Possible relationship to vasodilator function?. J. Anat..

[92] Dora K.A., Gallagher N.T., McNeish A., Garland C.J. (2008). Modulation of endothelial cell KCa3.1 channels during endothelium-derived hyperpolarizing factor signaling in mesenteric resistance arteries. Circ. Res..

[93] Coleman H.A., Tare M., Parkington H.C. (2017). Nonlinear effects of potassium channel blockers on endothelium-dependent hyperpolarization. Acta Physiol..

[94] Chen G.F., Suzuki H. (1990). Calcium dependency of the endothelium-dependent hyperpolarization in smooth muscle cells of the rabbit carotid artery. J. Physiol..

[95] Fukao M., Hattori Y., Kanno M., Sakuma I., Kitabatake A. (1997). Sources of Ca^2+^ in relation to generation of acetylcholine-induced endothelium-dependent hyperpolarization in rat mesenteric artery. Br. J. Pharmacol..

[96] Boudaka A., Al-Suleimani M., BaOmar H., Al-Lawati I., Zadjali F. (2016). Impairment of transient receptor potential Vanilloid 4-Mediated dilation in Mesenteric arteries of spontaneously hypertensive rats. Proc. Physiol. Soc..

[97] Zhang D.X., Mendoza S.A., Bubolz A.H., Mizuno A., Ge Z.D., Li R., Warltier D.C., Suzuki M., Gutterman D.D. (2009). Transient receptor potential vanilloid type 4-deficient mice exhibit impaired endothelium-dependent relaxation induced by acetylcholine in vitro and in vivo. Hypertension.

[98] Gao F., Sui D., Garavito R.M., Worden R.M., Wang D.H. (2009). Salt intake augments hypotensive effects of transient receptor potential vanilloid 4: Functional significance and implication. Hypertension.

[99] Nishijima Y., Zheng X., Lund H., Suzuki M., Mattson D.L., Zhang D.X. (2014). Characterization of blood pressure and endothelial function in TRPV4-deficient mice with l-NAME- and angiotensin II-induced hypertension. Physiol. Rep..

[100] He D., Pan Q., Chen Z., Sun C., Zhang P., Mao A., Zhu Y., Li H., Lu C., Xie M. (2017). Treatment of hypertension by increasing impaired endothelial TRPV4-KCa2.3 interaction. EMBO Mol. Med..

[101] Rath G., Dessy C., Feron O. (2009). Caveolae, caveolin and control of vascular tone: Nitric oxide (NO) and endothelium derived hyperpolarizing factor (EDHF) regulation. J. Physiol. Pharmacol..

[102] Haddy F.J., Vanhoutte P.M., Feletou M. (2006). Role of potassium in regulating blood flow and blood pressure. Am. J. Physiol. Regul. Integr. Comp. Physiol..

[103] McCarron J.G., Halpern W. (1990). Impaired potassium-induced dilation in hypertensive rat cerebral arteries does not reflect altered Na+,K(+)-ATPase dilation. Circ. Res..

[104] Chrissobolis S., Sobey C.G. (2003). Inwardly rectifying potassium channels in the regulation of vascular tone. Curr. Drug Targets.

[105] Overbeck H.W., Derifield R.S., Pamnani M.B., Sözen T. (1974). Attenuated vasodilator responses to K^+^ in essential hypertensive men. J. Clin. Investig..

[106] Quayle J.M., Nelson M.T., Standen N.B. (1997). ATP-sensitive and inwardly rectifying potassium channels in smooth muscle. Physiol. Rev..

[107] Hill C.E. (2008). Inward rectification and vascular function: As it was in the beginning. J. Physiol..

[108] Doughty J.M., Boyle J.P., Langton P.D. (2001). Blockade of chloride channels reveals relaxations of rat small mesenteric arteries to raised potassium. Br. J. Pharmacol..

[109] Crane G.J., Walker S.D., Dora K.A., Garland C.J. (2003). Evidence for a differential cellular distribution of inward rectifier K channels in the rat isolated mesenteric artery. J. Vasc. Res..

[110] Dora K.A., Garland C.J. (2001). Properties of smooth muscle hyperpolarization and relaxation to K^+^ in the rat isolated mesenteric artery. Am. J. Physiol. Heart Circ. Physiol..

[111] Sonkusare S.K., Dalsgaard T., Bonev A.D., Nelson M.T. (2016). Inward rectifier potassium (Kir2.1) channels as end-stage boosters of endothelium-dependent vasodilators. J. Physiol..

[112] Ahn S.J., Fancher I.S., Bian J.T., Zhang C.X., Schwab S., Gaffin R., Phillips S.A., Levitan I. (2017). Inwardly rectifying K^+^ channels are major contributors to flow-induced vasodilatation in resistance arteries. J. Physiol..

[113] Iddings J.A., Kim K.J., Zhou Y., Higashimori H., Filosa J.A. (2015). Enhanced parenchymal arteriole tone and astrocyte signaling protect neurovascular coupling mediated parenchymal arteriole vasodilation in the spontaneously hypertensive rat. J. Cereb. Blood Flow Metab..

[114] Longden T.A., Nelson M.T. (2015). Vascular inward rectifier K^+^ channels as external K^+^ sensors in the control of cerebral blood flow. Microcirculation.

[115] Tajada S., Cidad P., Moreno-Domínguez A., Pérez-García M.T., López-López J.R. (2012). High blood pressure associates with the remodelling of inward rectifier K^+^ channels in mice mesenteric vascular smooth muscle cells. J. Physiol..

[116] Goto K., Edwards F.R., Hill C.E. (2007). Depolarization evoked by acetylcholine in mesenteric arteries of hypertensive rats attenuates endothelium-dependent hyperpolarizing factor. J. Hypertens..

[117] Yang C., Kwan Y.W., Chan S.W., Lee S.M., Leung G.P. (2010). Potentiation of EDHF-mediated relaxation by chloride channel blockers. Acta Pharmacol. Sin..

[118] Jackson W.F. (2018). K_V_ channels and the regulation of vascular smooth muscle tone. Microcirculation.

[119] Nieves-Cintrón M., Syed A.U., Nystoriak M.A., Navedo M.F. (2018). Regulation of voltage-gated potassium channels in vascular smooth muscle during hypertension and metabolic disorders. Microcirculation.

[120] Quignard J.F., Feletou M., Edwards G., Duhault J., Weston A.H., Vanhoutte P.M. (2000). Role of endothelial cell hyperpolarization in EDHF-mediated responses in the guinea-pig carotid artery. Br. J. Pharmacol..

[121] Sadanaga T., Ohya Y., Ohtsubo T., Goto K., Fujii K., Abe I. (2002). Decreased 4-aminopyridine sensitive K^+^ currents in endothelial cells from hypertensive rats. Hypertens. Res..

[122] Kitazono T., Heistad D.D., Faraci F.M. (1993). ATP-sensitive potassium channels in the basilar artery during chronic hypertension. Hypertension.

[123] Ohya Y., Setoguchi M., Fujii K., Nagao T., Abe I., Fujishima M. (1996). Impaired action of levcromakalim on ATP-sensitive K^+^ channels in mesenteric artery cells from spontaneously hypertensive rats. Hypertension.

[124] Ishizaka H., Kuo L. (1997). Endothelial, ATP-sensitive potassium channels mediate coronary microvascular dilation to hyperosmolarity. Am. J. Physiol..

[125] Aziz Q., Li Y., Anderson N., Ojake L., Tsisanova E., Tinker A. (2017). Molecular and functional characterization of the endothelial ATP-sensitive potassium channel. J. Biol. Chem..

[126] Rogers P.A., Dick G.M., Knudson J.D., Focardi M., Bratz I.N., Swafford A.N., Saitoh S., Tune J.D., Chilian W.M. (2006). H_2_O_2_-induced redox-sensitive coronary vasodilation is mediated by 4-aminopyridine-sensitive K^+^ channels. Am. J. Physiol. Heart Circ. Physiol..

[127] Lacza Z., Puskar M., Kis B., Perciaccante J.V., Miller A.W., Busija D.W. (2002). Hydrogen peroxide acts as an EDHF in the piglet pial vasculature in response to bradykinin. Am. J. Physiol. Heart Circ. Physiol..

[128] Cheang W.S., Wong W.T., Shen B., Lau C.W., Tian X.Y., Tsang S.Y., Yao X., Chen Z.Y., Huang Y. (2010). 4-aminopyridine-sensitive K^+^ channels contributes to NaHS-induced membrane hyperpolarization and relaxation in the rat coronary artery. Vascul. Pharmacol..

[129] Gao Y.J., Zhang Y., Hirota S., Janssen L.J., Lee R.M. (2004). Vascular relaxation response to hydrogen peroxide is impaired in hypertension. Br. J. Pharmacol..

[130] Meng G., Ma Y., Xie L., Ferro A., Ji Y. (2015). Emerging role of hydrogen sulfide in hypertension and related cardiovascular diseases. Br. J. Pharmacol..

[131] Greaney J.L., Kutz J.L., Shank S.W., Jandu S., Santhanam L., Alexander L.M. (2017). Impaired hydrogen sulfide-mediated vasodilation contributes to microvascular endothelial dysfunction in hypertensive adults. Hypertension.

[132] Large W.A., Wang Q. (1996). Characteristics and physiological role of the Ca(2+)-activated Cl^-^ conductance in smooth muscle. Am. J. Physiol..

[133] Yamamoto Y., Suzuki H. (2005). Dependency of endothelial cell function on vascular smooth muscle cells in guinea-pig mesenteric arteries and arterioles. J. Smooth Muscle Res..

[134] Groschner K., Graier W.F., Kukovetz W.R. (1994). Histamine induces K^+^, Ca^2+^, and Cl^-^ currents in human vascular endothelial cells. Role of ionic currents in stimulation of nitric oxide biosynthesis. Circ. Res..

[135] Nilius B., Prenen J., Szücs G., Wei L., Tanzi F., Voets T., Droogmans G. (1997). Calcium-activated chloride channels in bovine pulmonary artery endothelial cells. J. Physiol..

[136] Yamamoto Y., Suzuki H. (2007). Effects of increased intracellular Cl^-^ concentration on membrane responses to acetylcholine in the isolated endothelium of guinea pig mesenteric arteries. J. Physiol. Sci..

[137] Yang Y.D., Cho H., Koo J.Y., Tak M.H., Cho Y., Shim W.S., Park S.P., Lee J., Lee B., Kim B.M. (2008). TMEM16A confers receptor-activated calcium-dependent chloride conductance. Nature.

[138] Caputo A., Caci E., Ferrera L., Pedemonte N., Barsanti C., Sondo E., Pfeffer U., Ravazzolo R., Zegarra-Moran O., Galietta L.J. (2008). TMEM16A, a membrane protein associated with calcium-dependent chloride channel activity. Science.

[139] Schroeder B.C., Cheng T., Jan Y.N., Jan L.Y. (2008). Expression cloning of TMEM16A as a calcium-activated chloride channel subunit. Cell.

[140] Wang B., Li C., Huai R., Qu Z. (2015). Overexpression of ANO1/TMEM16A, an arterial Ca^2+^-activated Cl^-^ channel, contributes to spontaneous hypertension. J. Mol. Cell. Cardiol..

[141] Ma M.M., Gao M., Guo K.M., Wang M., Li X.Y., Zeng X.L., Sun L., Lv X.F., Du Y.H., Wang G.L. (2017). TMEM16A Contributes to Endothelial Dysfunction by Facilitating Nox2 NADPH Oxidase-Derived Reactive Oxygen Species Generation in Hypertension. Hypertension.

[142] Hutri-Kähönen N., Kähönen M., Tolvanen J.P., Wu X., Sallinen K., Pörsti I. (1997). Ramipril therapy improves arterial dilation in experimental hypertension. Cardiovasc. Res..

[143] Onaka U., Fujii K., Abe I., Fujishima M. (1998). Antihypertensive treatment improves endothelium-dependent hyperpolarization in the mesenteric artery of spontaneously hypertensive rats. Circulation.

[144] Kähönen M., Tolvanen J.P., Kalliovalkama J., Wu X., Karjala K., Mäkynen H., Pörsti I. (1999). Losartan and enalapril therapies enhance vasodilatation in the mesenteric artery of spontaneously hypertensive rats. Eur. J. Pharmacol..

[145] Goto K., Fujii K., Onaka U., Abe I., Fujishima M. (2000). Renin-angiotensin system blockade improves endothelial dysfunction in hypertension. Hypertension.

[146] Vettoretti S., Ochodnicky P., Buikema H., Henning R.H., Kluppel C.A., de Zeeuw D., van Dokkum R.P. (2006). Altered myogenic constriction and endothelium-derived hyperpolarizing factor-mediated relaxation in small mesenteric arteries of hypertensive subtotally nephrectomized rats. J. Hypertens..

[147] Seki T., Goto K., Kansui Y., Ohtsubo T., Matsumura K., Kitazono T. (2017). Angiotensin II Receptor-Neprilysin Inhibitor Sacubitril/Valsartan Improves Endothelial Dysfunction in Spontaneously Hypertensive Rats. J. Am. Heart Assoc..

[148] Goto K., Fujii K., Onaka U., Abe I., Fujishima M. (2000). Angiotensin-converting enzyme inhibitor prevents age-related endothelial dysfunction. Hypertension.

[149] Kansui Y., Fujii K., Goto K., Abe I., Iida M. (2002). Angiotensin II receptor antagonist improves age-related endothelial dysfunction. J. Hypertens..

[150] Albarwani S., Al-Siyabi S., Al-Husseini I., Al-Ismail A., Al-Lawati I., Al-Bahrani I., Tanira M.O. (2015). Lisinopril alters contribution of nitric oxide and K(Ca) channels to vasodilatation in small mesenteric arteries of spontaneously hypertensive rats. Physiol. Res..

[151] More A.S., Mishra J.S., Hankins G.D., Yallampalli C., Sathishkumar K. (2015). Enalapril normalizes endothelium-derived hyperpolarizing factor-mediated relaxation in mesenteric artery of adult hypertensive rats prenatally exposed to testosterone. Biol. Reprod..

[152] Toro L., Amador M., Stefani E. (1990). ANG II inhibits calcium-activated potassium channels from coronary smooth muscle in lipid bilayers. Am. J. Physiol..

[153] Minami K., Hirata Y., Tokumura A., Nakaya Y., Fukuzawa K. (1995). Protein kinase C-independent inhibition of the Ca(2+)-activated K^+^ channel by angiotensin II and endothelin-1. Biochem. Pharmacol..

[154] Zhao L.M., Wang Y., Ma X.Z., Wang N.P., Deng X.L. (2014). Advanced glycation end products impair K(Ca)3.1- and K(Ca)2.3-mediated vasodilatation via oxidative stress in rat mesenteric arteries. Pflugers. Arch..

[155] Kaw S., Hecker M. (1999). Endothelium-derived hyperpolarizing factor, but not nitric oxide or prostacyclin release, is resistant to menadione-induced oxidative stress in the bovine coronary artery. Naunyn. Schmiedebergs. Arch. Pharmacol..

[156] Hamilton C.A., McPhaden A.R., Berg G., Pathi V., Dominiczak A.F. (2001). Is hydrogen peroxide an EDHF in human radial arteries?. Am. J. Physiol. Heart Circ. Physiol..

[157] Louis W.J., Howes L.G. (1990). Genealogy of the spontaneously hypertensive rat and Wistar-Kyoto rat strains: Implications for studies of inherited hypertension. J. Cardiovasc. Pharmacol..

[158] Matthaei K.I. (2009). Identification of therapeutic drug targets through genetically manipulated mice: Are we getting it right?. Pharmacol. Ther..

[159] Bellien J., Thuillez C., Joannides R. (2008). Contribution of endothelium-derived hyperpolarizing factors to the regulation of vascular tone in humans. Fundam. Clin. Pharmacol..

[160] Taddei S., Ghiadoni L., Virdis A., Buralli S., Salvetti A. (1999). Vasodilation to bradykinin is mediated by an ouabain-sensitive pathway as a compensatory mechanism for impaired nitric oxide availability in essential hypertensive patients. Circulation.

[161] Taddei S., Versari D., Cipriano A., Ghiadoni L., Galetta F., Franzoni F., Magagna A., Virdis A., Salvetti A. (2006). Identification of a cytochrome P450 2C9-derived endothelium-derived hyperpolarizing factor in essential hypertensive patients. J. Am. Coll. Cardiol..

[162] Sainsbury C.A., Coleman J., Brady A.J., Connell J.M., Hillier C., Petrie J.R. (2007). Endothelium-dependent relaxation is resistant to inhibition of nitric oxide synthesis, but sensitive to blockade of calcium-activated potassium channels in essential hypertension. J. Hum. Hypertens..

[163] Li J., Zhou Z., Jiang D.J., Li D., Tan B., Liu H., Li Y.J. (2007). Reduction of NO- and EDHF-mediated vasodilatation in hypertension: Role of asymmetric dimethylarginine. Clin. Exp. Hypertens..

[164] Kenny L.C., Baker P.N., Kendall D.A., Randall M.D., Dunn W.R. (2002). Differential mechanisms of endothelium-dependent vasodilator responses in human myometrial small arteries in normal pregnancy and pre-eclampsia. Clin. Sci..

[165] Luksha L., Luksha N., Kublickas M., Nisell H., Kublickiene K. (2010). Diverse mechanisms of endothelium-derived hyperpolarizing factor-mediated dilatation in small myometrial arteries in normal human pregnancy and preeclampsia. Biol. Reprod..

[166] Goulopoulou S. (2017). Maternal Vascular Physiology in Preeclampsia. Hypertension.

[167] Choi S., Kim J.A., Na H.Y., Kim J.E., Park S., Han K.H., Kim Y.J., Suh S.H. (2013). NADPH oxidase 2-derived superoxide downregulates endothelial KCa3.1 in preeclampsia. Free Radic. Biol. Med..

[168] Deja M.A., Gołba K.S., Widenka K., Mrozek R., Biernat J., Kolowca M., Malinowski M., Woś S. (2005). Angiotensin-converting enzyme inhibitors reveal non-NO-, non-prostacycline-mediated endothelium-dependent relaxation in internal thoracic artery of hypertensive patients. Int. J. Cardiol..

